# Differentiated mosquito adaptive strategies underlie regional dominance of three ECSA sub-lineages in chikungunya virus

**DOI:** 10.1371/journal.ppat.1014677

**Published:** 2026-10-05

**Authors:** Yuhua Liao, Jingqi Yang, Jingzhe Shang, Hangyu Zhou, Aiping Wu

**Affiliations:** State Key Laboratory of Common Mechanism Research for Major Diseases, Suzhou Institute of Systems Medicine, Chinese Academy of Medical Sciences and Peking Union Medical College, Suzhou, Jiangsu, China; National and Kapodistrian University of Athens, GREECE

## Abstract

Chikungunya virus (CHIKV) has emerged as a rapidly expanding global health threat and has been reported in 119 countries and territories, with recent outbreaks across Asia, Europe, and the Americas. The East/Central/South African (ECSA) genotype has increasingly predominated in multiple regions, yet the molecular basis of its competitive advantage remains unresolved. Here, we demonstrate that three ECSA sub-lineages—ECSA-IOL, ECSA-2.1, and ECSA-2.2—have independently evolved distinct vector-associated evolutionary patterns that underlie their divergent geographic dominance. Using comparative phylogenomic analyses of complete CHIKV genomes and selection pressure analyses (RELAX/BUSTED/Contrast-FEL), we found that ECSA-2.1 showed intensified selection on structural-protein genes and accumulated substitutions previously associated with *Aedes aegypti*, explaining its rapid displacement of the Asian genotype across the Americas. In contrast, ECSA-IOL underwent adaptation strategy switching from *Aedes albopictus* to *Ae. aegypti*, maintaining its dominance across South and Southeast Asia, while ECSA-2.2 convergently acquired *Ae. albopictus* adaptation with additional envelope protein mutations, suggesting enhanced adaptive potential. Genome-wide analysis revealed lineage-characteristic and convergent substitutions, providing molecular signatures associated with ECSA competitive advantage. These findings suggest vector niche partitioning as an important driver of CHIKV global expansion dynamics, with potential implications for outbreak forecasting, vector control prioritization, and the design of broadly protective vaccines.

## Author summary

Chikungunya virus has spread to 119 countries and territories, with the East/Central/South African (ECSA) genotype now dominating globally. However, the evolutionary mechanisms driving this success have remained unclear. We analyzed over 4,000 viral genomes using comparative phylogenomic and selection-pressure analyses to uncover the hidden drivers of ECSA’s expansion. We discovered that three ECSA sub-lineages have each evolved distinct strategies to adapt to different mosquito species. ECSA-2.1 displaced the Asian genotype in the Americas by intensifying selection on structural-protein genes and fixing *Aedes aegypti*-adaptive mutations, while ECSA-IOL strategically switched its adaptation from *Aedes albopictus* to *Ae. aegypti* to dominate South and Southeast Asia. ECSA-2.2 acquired unique mutations favoring *Ae. albopictus* and recently emerged in regions where this mosquito is prevalent. Remarkably, vector niche partitioning—not simply faster evolutionary rates—explains why ECSA lineages outcompete other lineages. Our findings establish molecular signatures for tracking variant emergence and identify specific mutations as targets for future functional studies and vaccine development.

## Introduction

Chikungunya virus (CHIKV) is an alphavirus transmitted by *Aedes* mosquitoes, causing an acute febrile illness characterized by sudden high fever, rash, and severe polyarthralgia [1,2]. Unlike other arboviruses, some patients develop chronic arthritic symptoms persisting for months or even years following the acute phase [3,4]. Although case fatality rates are low, large-scale outbreaks impose substantial socioeconomic burdens. A resurgent outbreak on Réunion Island in 2024–2025 resulted in over 54,000 confirmed cases [5], while Guangdong Province, China, experienced its largest documented chikungunya outbreak in 2025 with more than 10,000 cases [6]. Autochthonous transmission was also documented in mainland France and Italy during the 2025 mosquito season [7–9], and epidemic activity continued across South and Southeast Asia [10,11]. In the Americas, CHIKV has caused an estimated 3.7 million suspected and laboratory-confirmed cases across 50 countries or territories since its introduction in 2013 [12] (all abbreviations are listed in S1 Table). No specific antiviral therapy exists, and although chikungunya vaccines have received regulatory approval in several countries, their availability and use remain limited [13]. Understanding the molecular basis of CHIKV spread requires examining its genome organization. CHIKV has an approximately 11.8-kb positive-sense, single-stranded RNA genome. The 5′ region encodes four nonstructural proteins (nsP1–4) forming the replication complex, while the 3′ region encodes five structural proteins (C, E3, E2, 6K and E1) responsible for virion assembly [1]. E2 and E1 form heterodimers constituting envelope spikes [14], E2 mediates receptor binding [15], E1 mediates membrane fusion and contains key mosquito adaptation sites [16,17], and their co-evolution is critical for viral transmission efficiency [18].

Since its initial isolation in 1952, CHIKV has transitioned from localized African circulation to global epidemic spread [2,19]. The 2004–2006 Indian Ocean outbreak marked a turning point [20], with the E1:A226V mutation increasing viral infectivity and transmission in *Ae. albopictus* [16]; in 2013, autochthonous CHIKV transmission was first detected in the Americas, establishing sustained epidemic transmission in the Americas [21,22]. CHIKV exhibits substantial genotypic diversity [23]. The West African genotype remains confined to sylvatic cycles, while the Asian genotype initiated the 2013–2015 American outbreak [21,24]; and the ECSA genotype is currently the dominant lineage globally, has diversified into ECSA-IOL, ECSA-2, and other sub-lineages [5,25]. Recent surveillance data suggest ongoing lineage turnover, with ECSA appearing to displace other genotypes in multiple regions, yet the molecular evolutionary mechanisms driving these lineage dynamics remain poorly understood.

Previous studies have primarily examined functional effects of individual adaptive mutations, such as E1:A226V enhancing *Ae. albopictus* adaptation, and E2:V264A and E1:K211E/T associated with *Ae. aegypti* adaptation [16,26,27]. However, these studies largely focused on functional validation of individual mutations, with systematic comparisons of lineage-level molecular features and vector adaptation strategies remaining limited. The two primary CHIKV vectors, *Ae. aegypti* and *Ae. albopictus,* have markedly different global distributions [28]: *Ae. aegypti* is concentrated primarily in tropical and subtropical urban areas, while *Ae. albopictus* has extensively expanded into temperate regions in recent decades, with its cold tolerance potentially facilitating CHIKV transmission at higher latitudes [29]. Viral adaptation capacity to different vectors may determine epidemic potential in specific geographic regions, but whether ECSA sub-lineages employ distinct vector adaptation strategies has not been systematically investigated.

These observations raise an important and broader question, whether arbovirus adaptive evolution follows predictable patterns [30]. Establishing transmission in new environments requires viruses to adapt to both vertebrate hosts and arthropod vectors, yet how this dual adaptive pressure shapes viral genome evolution remains unclear. Specifically, the mechanisms underlying ECSA’s global displacement of other genotypes, and whether its sub-lineages employ distinct adaptive strategies, remain unclear. Through comparative evolutionary genomics integrating phylogenetic analysis, molecular clock analysis, selection analyses (RELAX/BUSTED/Contrast-FEL), and mosquito adaptation site tracking, this study systematically addresses the following questions: (1) What sub-lineage structure exists within the ECSA genotype, and how are these sub-lineages geographically distributed? (2) Do these sub-lineages exhibit distinct vector-associated evolutionary patterns? (3) How do these patterns contributed to regional lineage predominance? By addressing these questions, we establish a molecular evolutionary framework explaining how three ECSA sub-lineages achieve regional dominance through distinct vector adaptations. These findings provide insights for arbovirus evolution research and molecular epidemiological surveillance.

## Results

### Four historical phases of CHIKV global transmission and the evolutionary rate paradox

Based on WHO surveillance reports and chikungunya fever epidemiological history [31], we divided the history of CHIKV transmission into four phases (Fig 1A). Phase 1 (P1, 1952–2003) represented the early sporadic Africa-Asia transmission, with the African region as the primary endemic area (64.3%). Phase 2 (P2, 2004–2012) marked the onset of the Indian Ocean epidemic, accompanied by the E1:A226V mutation and expansion to five WHO regions. Phase 3 (P3, 2013–2016) witnessed transmission pattern restructuring. Among 61 newly affected countries, the Americas region (AMRO/PAHO) dominated (77.0%), reporting approximately 1.8 million suspected cases. Phase 4 (P4, 2017–2025) exhibited a global multi-center epidemic pattern, with CHIKV distribution extending to 119 countries and territories.

**Fig 1:**
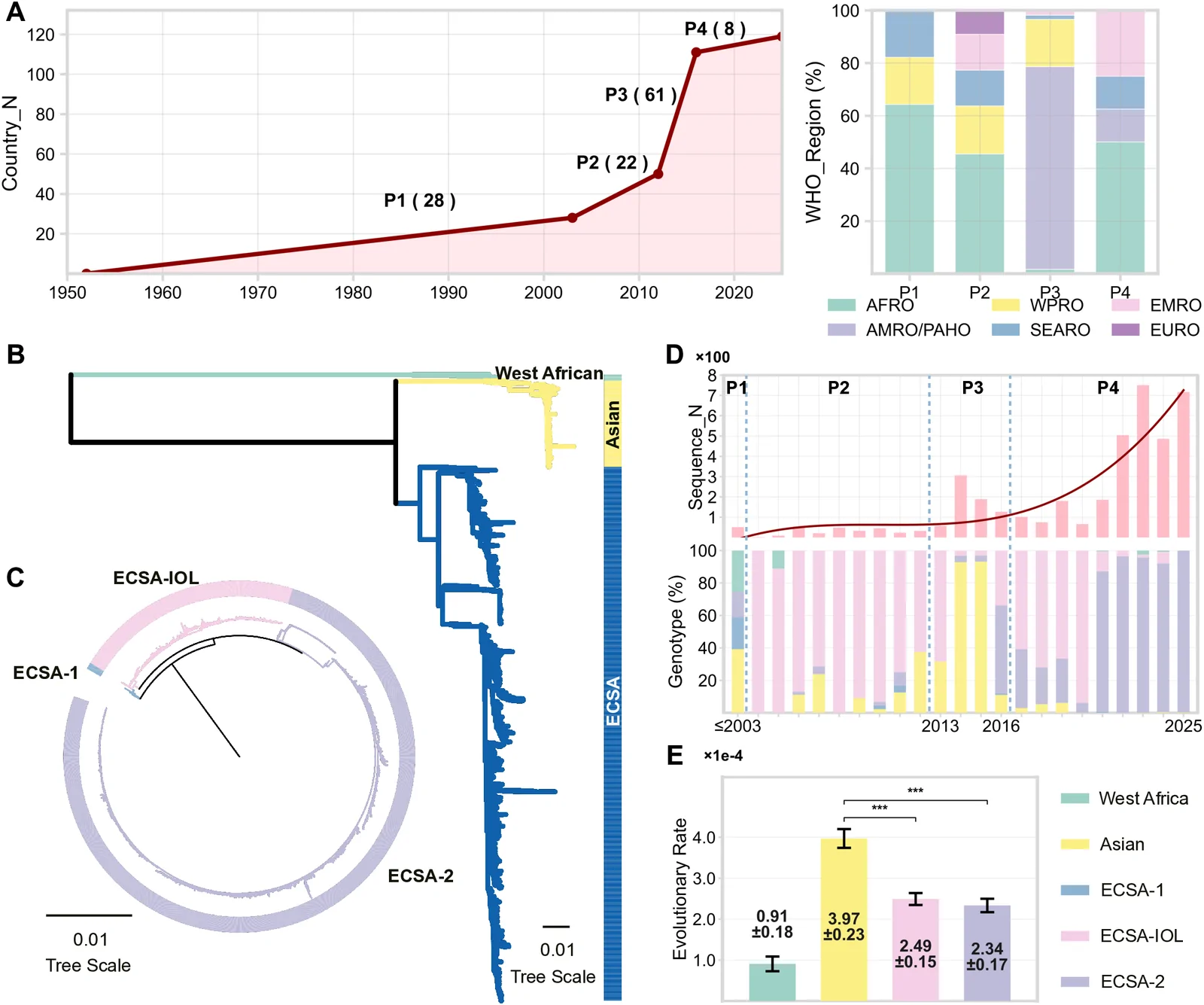
Global CHIKV transmission history and genotype-specific evolutionary rates. **(A)** Four phases of CHIKV global transmission. P1 (1952–2003): early Africa-Asia sporadic transmission; P2 (2004–2012): Indian Ocean outbreak with E1:A226V emergence; P3 (2013–2016): Americas transmission restructuring; P4 (2017–2025): global multi-center epidemic. Numbers indicate newly affected countries per period; stacked bars show WHO regional distribution (S2 Data). **(B)** Maximum-likelihood phylogenetic tree based on 4,120 whole-genome sequences. Branch colors: West African (green), Asian (yellow), ECSA (dark blue). The O’nyong-nyong virus was used as the outgroup. **(C)** Circular phylogeny showing subdivision of the ECSA genotype into the ECSA-1, ECSA-IOL, and ECSA-2 sub-lineages. **(D)** Sequence counts and genotype frequency dynamics. Top: sequence counts over time; bottom: stacked bar plot showing succession from multi-genotype co-circulation to ECSA dominance during 1952–2025. **(E)** TreeTime-estimated evolutionary rates by genotype. Bars show nucleotide substitution-rate estimates (substitutions per site per year). Error bars indicate the one-standard-deviation uncertainties reported by TreeTime. Pairwise differences were assessed using two-sided z-tests with Bonferroni correction across six comparisons: ***adjusted *P* < 0.001. ECSA-1 was not included owing to the small sample (n = 22) and insufficient temporal signal.

Phylogenetic analysis of 4,120 complete CHIKV genomes resolved three major genotypes—West African, Asian, and ECSA—with the ECSA genotype further subdivided into ECSA-1, ECSA-IOL, and ECSA-2 (Fig 1B and 1C). Temporal analysis showed (Fig 1D) that P1 was characterized by multi-genotype co-circulation; P2 by a rapid rise in ECSA-IOL; P3 by expansion in the Americas followed by replacement of the Asian genotype by ECSA; P4 by ECSA dominance, with a shift from ECSA-IOL to ECSA-2, with ECSA-2 sub-lineages predominating among sampled genomes from the 2024–2025 outbreaks in Réunion, Guangdong, and the Americas.

Molecular clock analysis revealed an unexpected pattern (Fig 1E). The Asian genotype exhibited the highest evolutionary rate (3.97 ± 0.23 × 10^-4^ substitutions/site/year), significantly exceeding (*P* < 0.001) those of ECSA-IOL (2.49 ± 0.15 × 10^-4^) and ECSA-2 (2.34 ± 0.17 × 10^-4^), whereas the West African genotype showed the lowest rate (0.91 ± 0.18 × 10^-4^). Yet the fastest-evolving Asian genotype was displaced by slower-evolving ECSA lineages. This apparent discordance suggests that nucleotide substitution rate alone does not explain ECSA lineage success.

### ECSA-2 subdivision and sub-lineage distribution patterns

To elucidate the factors underlying ECSA expansion, we analyzed the spatiotemporal dynamics of each sub-lineage. We subsampled the complete-genome dataset by genotype, sampling date, and geographic location to construct a Bayesian time-scaled phylogeny comprising 726 sequences (Fig 2A). Phylogenetic analysis resolved ECSA-2 into two distinct sub-lineages with maximum posterior support (PP = 1.0), with their divergence dated to July 1954 (95% HPD: August 1948–February 1958; all node divergence-time estimates are shown in S1 Fig). ECSA-2.1 was distributed almost entirely in the Americas, while ECSA-2.2 was distributed in Africa early, with a recent 2024–2025 outbreak cluster within ECSA-2.2 (tMRCA January 2023, 95% HPD: November 2021–October 2023) mainly distributed in Réunion, China, and some European countries (including France and Italy). ECSA-IOL was mainly distributed in Asia.

**Fig 2:**
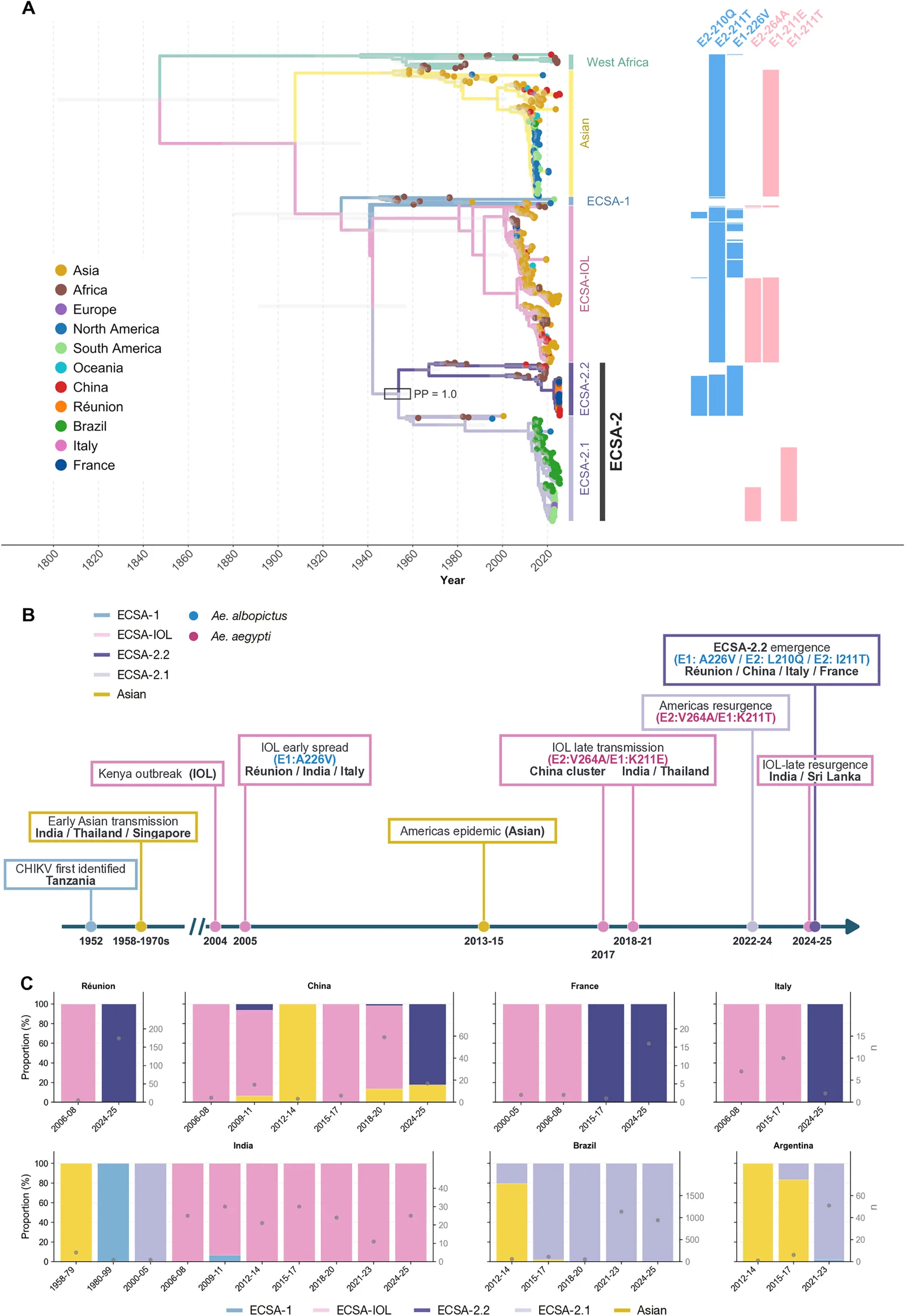
ECSA-2 subdivision and regional genotype dynamics. **(A)** Bayesian time-scaled phylogeny based on 726 sequences. Branch colors indicate genotypes; node circles indicate continental origin, with recent outbreak locations (China, Réunion, Brazil, France, and Italy) highlighted. Right heatmap shows six experimentally validated vector-adaptive amino acid substitutions: *Ae. albopictus*–adaptive substitutions (blue: E2:L210Q, E2:I211T, E1:A226V) and *Ae. aegypti* adaptation sites (pink: E2:V264A, E1:K211E, E1:K211T). ECSA-2.1/ECSA-2.2 split node (PP = 1.0), divergence time 1954 (95% HPD: 1948–1958). **(B)** Schematic timeline of major genotype transitions and associated vector-adaptive mutations across successive outbreak waves. Blue dots mark *Ae. albopictus*–associated sites; magenta dots mark *Ae. aegypti*–associated sites. **(C)** Genotype dynamics in seven representative locations. Stacked bar plots showing genotype frequency changes in Réunion, China, France, Italy, India, Brazil, and Argentina (gray points indicate sample size), showing the regional predominance patterns of the three ECSA sub-lineages.

The time tree displays six experimentally validated vector-adaptive amino acid substitutions, colored by vector type (Fig 2A). These include three *Ae. albopictus* adaptation substitutions [16,18,32] (E2:L210Q, E2:I211T, and E1:A226V) and three *Ae. aegypti* adaptation substitutions [26,27] (E2:V264A, E1:K211E, and E1:K211T). The heatmap shows E1:A226V emerged independently in early IOL and ECSA-2.2, while the E2:V264A/E1:K211E combination mainly appeared in late IOL, whereas E2:V264A and E1:K211T were also inferred to have emerged independently in ECSA-2.1, consistent with convergent evolution. We also visualized geographic distribution and these key mutations in the complete phylogenetic tree (S2 Fig).

Major outbreak events were associated with distinct lineage-specific vector-adaptive substitutions (Fig 2B). ECSA-IOL expanded from Kenya to Réunion, India, and Italy during 2004–2007 in association with E1:A226V. Later ECSA-IOL transmission in Asia during 2017–2021 was characterized by E1:K211E and E2:V264A. The Asian genotype caused epidemics in the Americas during 2013–2015, followed by the expansion of ECSA-2.1 carrying E1:K211T and E2:V264A during 2022–2024. In 2024–2025, a recent ECSA-2.2 cluster carrying E1:A226V, E2:L210Q, and E2:I211T was detected in Réunion, China, Italy, and France. During the same period, ECSA-IOL resurged in India and Sri Lanka.

Regional genotype trajectories revealed distinct succession patterns (Fig 2C). India experienced multiple early genotype replacements, but ECSA-IOL has predominated since the 2005–2006 Indian Ocean epidemic. Réunion, France, and Italy shifted from ECSA-IOL to ECSA-2.2. China underwent repeated genotype turnover, with ECSA-2.2 predominating in 2024–2025. Brazil and Argentina shifted from the Asian genotype to ECSA-2.1. Together, these patterns suggest region-specific epidemiological success of the three ECSA sub-lineages, with ECSA-IOL predominating in South Asia, ECSA-2.1 in South America, and ECSA-2.2 in the Indian Ocean region, China, and parts of Europe.

### Characteristic mutation accumulation and convergent evolution among three ECSA sub-lineages

To characterize ECSA sub-lineage divergence, we extracted 3,512 ECSA sequences for comparative analysis (Fig 3A). Analysis of 2024–2025 circulating strains showed all three sub-lineages contributing to ongoing transmission in different regions. Whole-genome comparison revealed differences in mutation load among sub-lineages (Fig 3B). After excluding sites conserved in Asian and West African genotypes, ECSA-IOL accumulated 20 lineage-characteristic substitutions, ECSA-2.1 had 22, while ECSA-2.2 exhibited the highest mutation load with 49 characteristic mutations (S2 Table). Four sites are shared among all three sub-lineages: nsP2:H374Y, nsP3:I376T, nsP4:Q500L, and E2:A164T, each with fixation frequency >90%. These sites are visualized in S3 Fig and are broadly fixed across ECSA.

**Fig 3:**
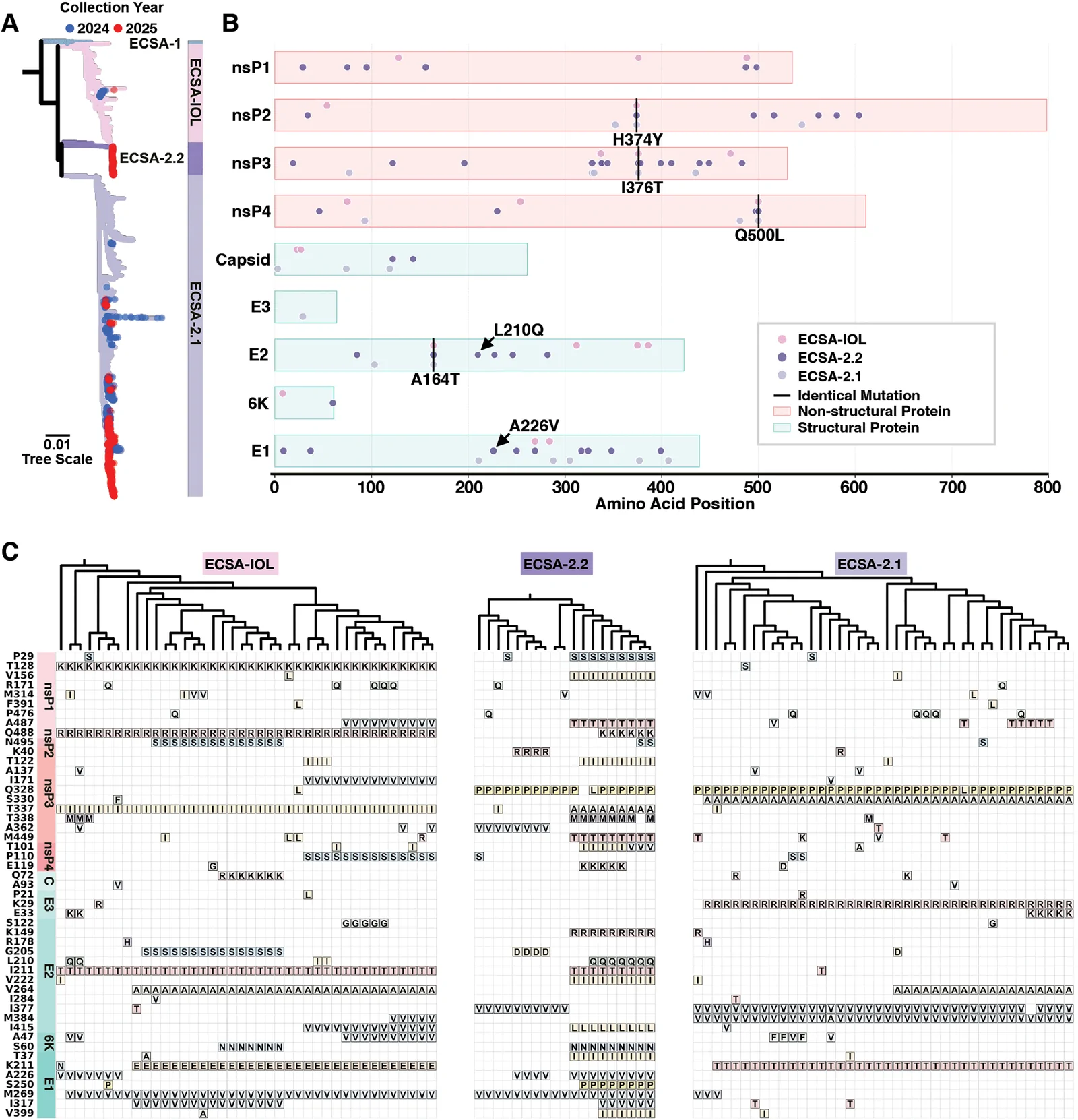
Lineage-characteristic substitutions and convergent sites in ECSA. **(A)** Distribution of sequences collected during 2024–2025 within the dataset of 3,512 ECSA genomes, showing that all three sub-lineages were represented among recently sampled genomes. **(B)** Genomic distribution of sub-lineage characteristic mutations. Scatter plot showing characteristic mutations along amino acid positions: ECSA-IOL (pink), ECSA-2.2 (dark purple), ECSA-2.1 (light purple). Black vertical lines mark four shared core adaptive sites (nsP2:H374Y, nsP3:I376T, nsP4:Q500L, E2:A164T; each occurring at a frequency of >90% in all three sub-lineages). ECSA-2.2 shows highest mutation load (49), followed by ECSA-2.1 (22) and ECSA-IOL (20). **(C)** Heatmap of 49 convergent sites. Rows represent convergent sites (organized by protein); columns represent sequences (grouped by sub-lineage).

To distinguish adaptive fixation from drift, we identified 49 convergent sites among the three sub-lineages (Fig 3C), distributed across the entire genome: E2 with the most (12), followed by nsP3 (10), nsP1 (9), E1 (7), nsP4 and E3 (3 each), C and 6K (2 each), and nsP2 (1). Notably, all six validated mosquito adaptation sites (E2:L210Q, E2:I211T, E1:A226V, E2:V264A, E1:K211E, E1:K211T) appeared among these convergent sites. Of these, we identified 25 sub-lineage-characteristic substitutions records at these convergent sites (23 unique amino acid substitutions; S3 Table), including key mosquito adaptation sites E2:210, E2:211, and E1:226, suggesting that mosquito adaptation represents a shared selective pressure across ECSA sub-lineages.

### ECSA-2.1 displaced the Asian genotype through enhanced structural protein selection and *Ae. aegypti* adaptation

Molecular clock analysis showed the American ECSA-2.1 outbreak cluster originated around March 2012 (95% HPD: April 2007–August 2013) (Fig 4A), representing a single ECSA introduction to the Americas. We extracted sequences exclusively from South America to reconstruct the molecular clock phylogeny, revealing temporal genotype replacement patterns (S4A Fig). The Asian genotype had been the dominant circulating lineage locally since 2013, while ECSA-2.1 frequency surged from approximately 4% in 2014–2015 to 91% in 2016, reaching near-fixation (>99%) by 2017, completing replacement within approximately two years (S4B Fig).

**Fig 4:**
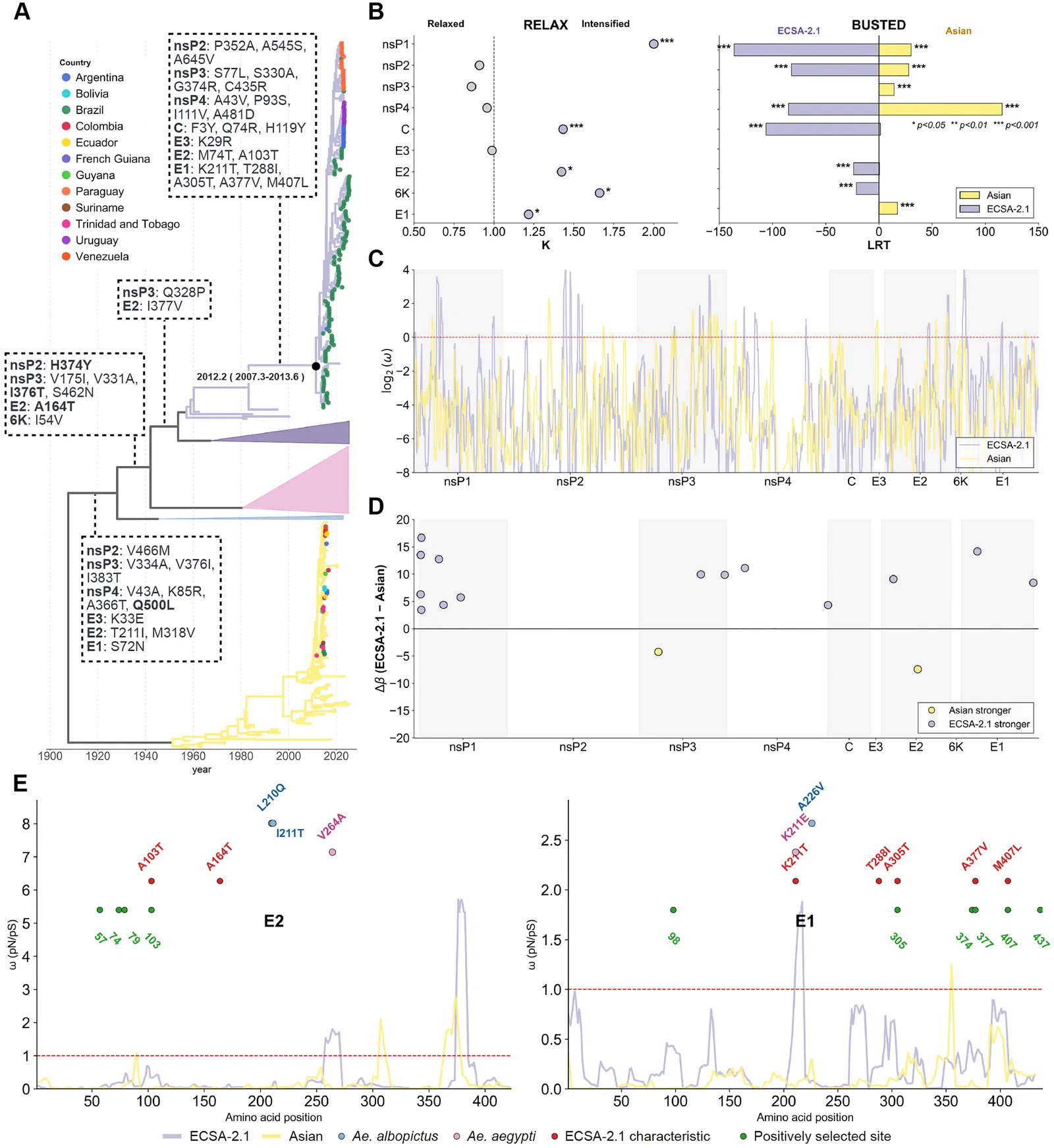
Selection pressure comparison between ECSA-2.1 and the Asian genotype in the Americas. **(A)** Molecular clock analysis of Americas ECSA-2.1 outbreak cluster. Time-scaled phylogeny showing the ECSA-2.1 outbreak cluster in the Americas, with an estimated MRCA in March 2012 (95% HPD: April 2007–August 2013). ECSA-2.1 branches are shown in purple and Asian genotype branches in yellow. Branches are annotated with amino acid substitutions identified by ancestral sequence reconstruction. **(B)** RELAX and BUSTED analyses. Left: RELAX showing ECSA-2.1 experienced intensified selection (*K* > 1) in nsP1 and multiple structural proteins relative to Asian. Right: BUSTED showing Asian positive selection concentrated in nonstructural proteins and E1, while ECSA-2.1 structural protein positive selection covers C, E2, and 6K. Asterisks indicate statistical significance: *P < 0.05, **P < 0.01, ***P < 0.001. **(C)** Whole-genome sliding window pN/pS analysis. Line plot showing log_2_(pN/pS) distribution along the genome for Asian (yellow) and ECSA-2.1 (purple). ECSA-2.1 showed elevated pN/pS in multiple regions of nsP1–3 and E2, 6K, E1. **(D)** Contrast-FEL site-level analysis. Δβ (ECSA-2.1 − Asian) indicates selection pressure differences. Positive Δβ values indicate stronger selection in ECSA-2.1, whereas negative values indicate stronger selection in the Asian genotype. After FDR correction (*Q* < 0.05), 16 sites significant: 14 with stronger ECSA-2.1 selection, 2 with stronger Asian selection. **(E)** Magnified sliding window analysis of E2 and E1. Four annotation categories integrated: *Ae. albopictus* adaptation sites (light blue), *Ae. aegypti* adaptation sites (pink), lineage-characteristic substitutions (red), positive selection sites (green, ≥ 2/3 methods consensus). *Ae. aegypti*–adaptive substitutions mapped to regions with high pN/pS values. The genomic positions of *Ae. albopictus*–adaptive substitutions corresponded to regions with low pN/pS values, and these substitutions were absent from ECSA-2.1.

We next compared selection patterns between ECSA-2.1 and the Asian genotype using multiple methods to identify the molecular basis underlying this displacement. RELAX analysis showed intensified selection (*K* > 1) on ECSA-2.1 in nsP1 (*K* = 26.17, *P* < 0.001) and multiple structural proteins including C, E2, 6K, and E1, while nsP2–4 showed no significant differences (Fig 4B, left). BUSTED analysis revealed differentiated positive selection patterns, with the Asian genotype showing significant positive selection in all nonstructural proteins (nsP1–4) and E1, whereas ECSA-2.1 displayed positive selection in nsP1, nsP2, nsP4, and structural proteins C, E2, and 6K (Fig 4B, right). Sliding window pN/pS analysis corroborated this pattern, with ECSA-2.1 showing elevated values across multiple regions in nsP1–3 and structural proteins E2, 6K, and E1 (Fig 4C). Contrast-FEL analysis identified 128 sites with differential selection (*P* < 0.05; S4C Fig), with the Asian genotype showing stronger selection at 73 sites and ECSA-2.1 at 55 sites. After FDR correction (*Q* < 0.05), 16 sites retained significance, 14 of which showed stronger selection in ECSA-2.1, with 7 of these sites located in nsP1 (Fig 4D). This convergent evidence from independent methods suggests a redistribution of selection pressure from replication machinery toward virion assembly and transmission components.

Functional annotation of E2 and E1 revealed concordance among lineage-characteristic substitutions, positive selection sites, and elevated pN/pS regions (Fig 4E). In E2, positive selection consensus sites and lineage-characteristic substitutions broadly overlapped with elevated pN/pS regions, with site 103 carrying dual positive selection and lineage-characteristic evidence. Although the E2 350–400 region shows a pN/pS peak, sites in this region were detected by only a single method, not reaching consensus threshold. In E1, sites 305, 377, and 407 carried dual positive selection and lineage-characteristic evidence, similarly corresponding to elevated pN/pS regions. The *Ae. aegypti* adaptation sites E2:V264A and E1:K211T both mapped to high pN/pS regions within ECSA-2.1. Notably, E1:K211T has been experimentally confirmed to enhance *Ae. aegypti* adaptation. All three *Ae. albopictus* adaptation sites (E2:L210Q, E2:I211T, E1:A226V) were absent, consistent with the American ecological context where *Ae. aegypti* is the primary vector. Internal differentiation of ECSA-2.1 further supported this association: a sub-cluster that fixed E2:V264A spread beyond Brazil to Argentina and Paraguay, whereas the sub-cluster lacking this mutation remained geographically confined (S4D Fig). Additionally, the E2 C-terminal region showed elevated pN/pS in ECSA-2.1, suggesting an actively evolving region that warrants surveillance.

### ECSA-IOL vector-adaptation shift and convergent adaptation of ECSA-2.2

To examine ECSA-2.2 replacement of ECSA-IOL in several regions, we performed cluster sampling of ECSA sequences and constructed a time-scaled phylogeny (Fig 5A). Phylogenetic analysis revealed a temporal shift in vector-associated substitutions within ECSA-IOL: early IOL (marked by E1:A226V) acquired this mutation during Indian Ocean region (e.g., Réunion) outbreaks, adapting to the locally dominant *Ae. albopictus*, while late IOL spreading to Southeast Asia in recent years (marked by E2:V264A and E1:K211E) switched to *Ae. aegypti* adaptation. Concurrently, ECSA-2.2 independently acquired E1:A226V, representing convergent *Ae. albopictus* adaptation with early IOL. This convergent evolution may explain ECSA-2.2 replacement of ECSA-IOL in *Ae. albopictus* distribution areas like Réunion—when ECSA-IOL’s adaptive focus shifted toward *Ae. aegypti*, ECSA-2.2 carrying E1:A226V gained competitive advantage in *Ae. albopictus* regions.

**Fig 5:**
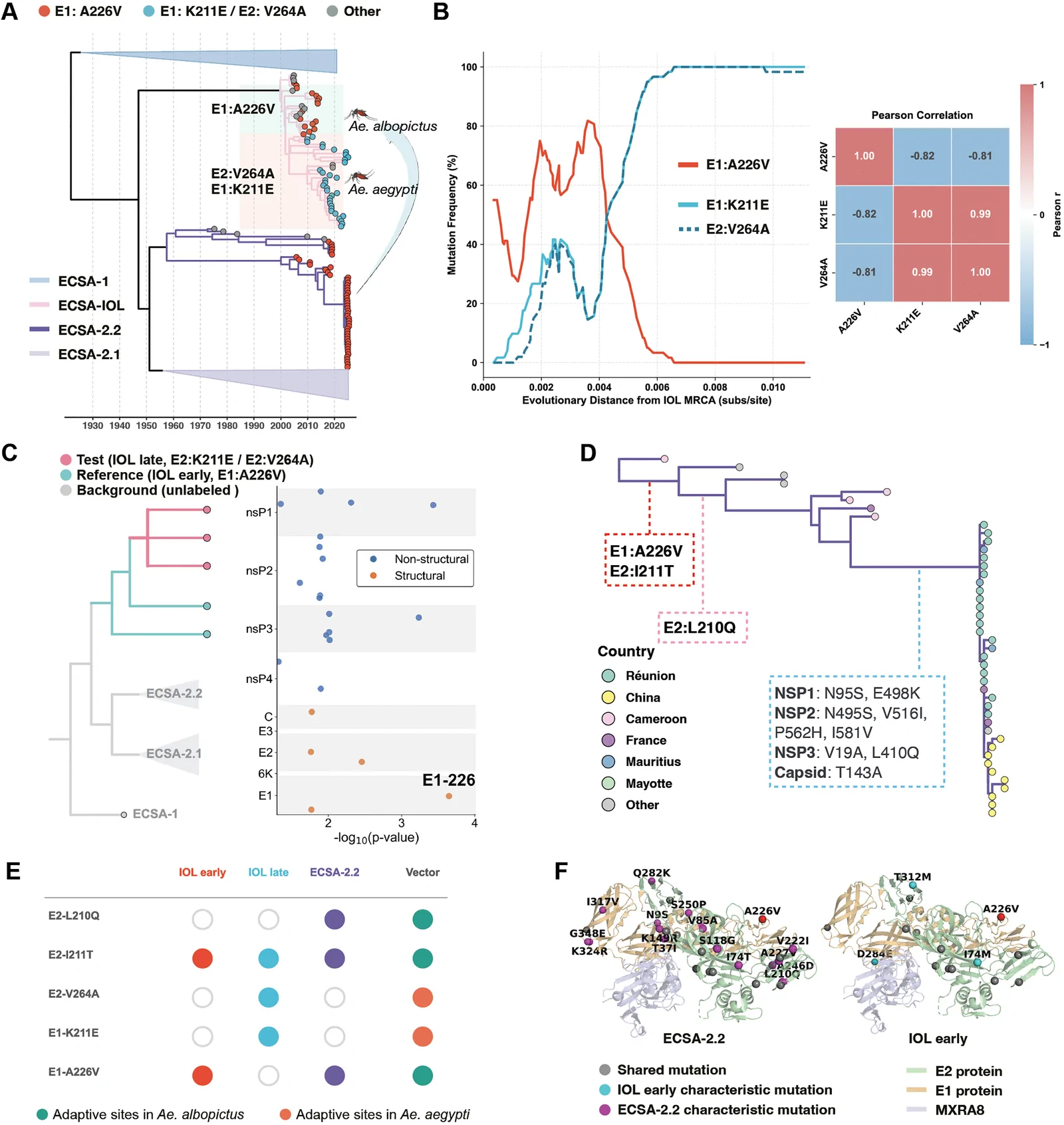
Vector adaptation dynamics in ECSA-IOL and ECSA-2.2. **(A)** Time-scaled phylogeny based on cluster-sampled ECSA sequences. Nodes colored by mosquito adaptation markers: E1:A226V (red), E1:K211E/E2:V264A (blue). Early IOL carried the *Ae. albopictus*–adaptive substitution E1:A226V, whereas late IOL carried the *Ae. aegypti*–adaptive substitutions E2:V264A and E1:K211E. ECSA-2.2 independently acquired E1:A226V through parallel evolution, representing convergent evolution with early IOL. **(B)** Mosquito adaptation site frequency dynamics. Left: sliding-window analysis showing E1:A226V (red) rising then declining, E2:V264A and E1:K211E (blue) increasing together. Right: correlation heatmap showing E2:V264A and E1:K211E strongly positively correlated (r = 0.99), E1:A226V negatively correlated with both. **(C)** Contrast-FEL analysis of early versus late IOL. Left: analysis framework with reference group (early IOL, cyan) vs. test group (late IOL, pink). Right: E1:226 shows most significant signal, with strong positive selection in early IOL but selection signal absent in late IOL. **(D)** Recent ECSA-2.2 outbreak cluster (ECSA-2.2) phylogeny. This lineage independently acquired E1:A226V, E2:I211T, and E2:L210Q (three experimentally validated *Ae. albopictus*–adaptive substitutions) along its ancestral branch, circulating in 2024–2025 in *Ae. albopictus*-dominant regions including Réunion and Guangdong. **(E)** Differential fixation patterns of mosquito adaptation sites across three lineages. Heatmap showing fixation status of five key sites in early IOL, late IOL, and ECSA-2.2. Cyan indicates *Ae. albopictus*–adaptive substitutions, and orange indicates *Ae. aegypti*–adaptive substitutions. **(F)** Structural mapping of envelope protein mutations. ECSA-2.2 and early IOL mutations mapped to E2–E1/MXRA8 complex (PDB ID: 6JO8). Gray indicates substitutions shared by early IOL and ECSA-2.2, and magenta indicates ECSA-2.2-specific substitutions.

Through sliding window analysis of all 758 ECSA-IOL sequences, we tracked frequency dynamics of three key mosquito adaptation sites along evolutionary distance (Fig 5B, left). E1:A226V rapidly rose to approximately 80% in early IOL then gradually declined, while E2:V264A and E1:K211E coordinately rose and approached fixation in late IOL. Correlation analysis showed association patterns among these three sites (Fig 5B, right). E2:V264A and E1:K211E were strongly positively correlated (*r* = 0.99), reported consistent with epistatic interaction, whereas E1:A226V was negatively correlated with both (*r* <−0.8), reflecting mutual exclusivity between *Ae. albopictus* and *Ae. aegypti* adaptation markers. Genome-wide, we identified 17 dynamic sites (mutation frequency change >75%; S5A Fig). 15 sites showed mutation frequency rising from approximately 0% to >85%, reflecting staged fixation of new mutations; 2 sites (E1:226 and nsP2:793) showed reversion trajectories. Correlation analysis divided these 17 sites into five co-mutation modules and several independent sites (S5B and S5C Fig): E2:V264A and E1:K211E constituted the core co-mutation pair for *Ae. aegypti* adaptation, other four modules involved coordinated changes in nonstructural and structural proteins; and E1:A226V, as an independent site, was negatively correlated with all co-mutation modules. Contrast-FEL analysis further revealed site-level selection pressure differences between early and late IOL (Fig 5C). Using early IOL (E1:A226V) as reference group and late IOL (E2:V264A/E1:K211E) as test group (Fig 5C, left), genome-wide scanning identified multiple differential selection sites (Fig 5C, right). The E1:226 site signal was most significant (*β =* 10.74, *P* < 0.001): this site showed strong positive selection in early IOL whereas the selection signal was not detected in late IOL, consistent with its frequency dynamic reversion trajectory.

As ECSA-IOL shifted toward *Ae. aegypti* adaptation, ECSA-2.2 independently acquired *Ae. albopictus*-associated substitutions and filled *Ae. albopictus’s* niche. Phylogenetic analysis showed (Fig 5D) that this lineage independently acquired E1:A226V (convergent with early IOL), together with E2:I211T and E2:L210Q on its trunk, all three experimentally confirmed to enhance *Ae. albopictus* adaptation. This lineage also accumulated a mutation set (nsP1:N95S/E498K, nsP2:N495S/V516I/P562H/I581V, nsP3:V19A/L410Q, and C:T143A) and circulated in Réunion, Guangdong, Mauritius, and France during 2024–2025, all regions where *Ae. albopictus* is the dominant vector. Differential fixation patterns of key mosquito adaptation sites across the three lineages further revealed this phenomenon (Fig 5E). Early IOL fixed *Ae. albopictus* adaptation sites E1:A226V and E2:I211T; late IOL switched to fixing *Ae. aegypti* adaptation sites E2:V264A and E1:K211E while E1:A226V did not reach fixation; ECSA-2.2 convergently acquired all three *Ae. albopictus* adaptation sites (E2:L210Q, E2:I211T, E1:A226V) but did not fix *Ae. aegypti* adaptation sites, consistent with its circulation pattern in *Ae. albopictus*-dominant regions. Mapping envelope protein mutations from early IOL and ECSA-2.2 onto the E2–E1/MXRA8 complex structure revealed multiple shared substitutions, located mainly in E2 (Fig 5F). ECSA-2.2-specific substitutions (magenta) were distributed across the envelope protein surface, highlighting candidate surface-exposed substitutions for further functional investigation.

To assess whether lineage-specific evolutionary patterns were associated with mosquito vector species, we examined 53 CHIKV genomes with explicit mosquito-source information, including 46 from four focal ECSA groups (S6A–S6C Fig). Early IOL and ECSA-2.2 were predominantly derived from *Ae. albopictus*, accounting for 6/8 and 7/8 genomes, respectively, whereas late IOL and ECSA-2.1 were predominantly derived from *Ae. aegypti*, accounting for 10/12 and 17/18 genomes. Vector-source composition differed significantly between early and late IOL (adjusted *P* = 0.019) and between ECSA-2.1 and ECSA-2.2 (adjusted *P* = 1.86 × 10^-4^). Among 18 literature-reported vector-associated substitutions (S3 Data), E1:A226V occurred mainly in early IOL and ECSA-2.2, E2:V264A and E1:K211E were concentrated in late IOL, and E1:K211T occurred mainly in ECSA-2.1. E2:L210Q was not detected in the 46 mosquito-derived genomes.

We further combined global mosquito occurrence data with CHIKV genomes containing sampling-country information to conduct an analysis stratified by sampling-country vector-occurrence class (S6D–S6F Fig). Globally, 63 countries had records of *Ae. aegypti* only, 33 had records of *Ae. albopictus* only, and 55 had records of both species (S6D Fig). Among key locations, occurrence records were dominated by *Ae. albopictus* in Réunion, China, France, and Italy and by *Ae. aegypti* in India and Argentina, whereas Brazil had large numbers of records for both species (S6E Fig). After excluding genomes sampled in countries with records of both vector species, 442 genomes remained. Lineage composition among the 442 genomes differed significantly between the two vector-occurrence classes (*P* < 0.001). Early IOL and ECSA-2.2 were enriched in *Ae. albopictus*-only countries, whereas ECSA-2.1 and the Asian genotype were enriched in *Ae. aegypti*-only countries (S6F Fig). Together, the mosquito-source and country-level analyses supported an association between CHIKV lineage distribution and vector ecology.

### Temporal dynamics of envelope protein evolution in emerging ECSA lineages

To explore patterns in ECSA adaptive evolution, we analyzed correlations between protein mutation proportions and molecular clock time in ECSA-2.1 and ECSA-2.2 (Fig 6A). The two lineages showed opposing temporal trends in E2 and E1 mutation proportions (Fig 6B): E2 (receptor-binding protein) mutation proportion decreased significantly over time (ECSA-2.1: r = −0.76, P < 0.001; ECSA-2.2: r = −0.57, P < 0.001), while E1 (fusion protein) mutation proportion increased significantly (ECSA-2.1: r = 0.51, P < 0.001; ECSA-2.2: r = 0.58, P < 0.001). For comparison, ECSA-IOL showed a weaker but consistent E1 rising trend (r = 0.40, P < 0.001), while Asian genotype showed the opposite pattern, with E1 mutation proportion decreasing significantly over time (r = −0.45, P < 0.001) (S7 Fig).

**Fig 6:**
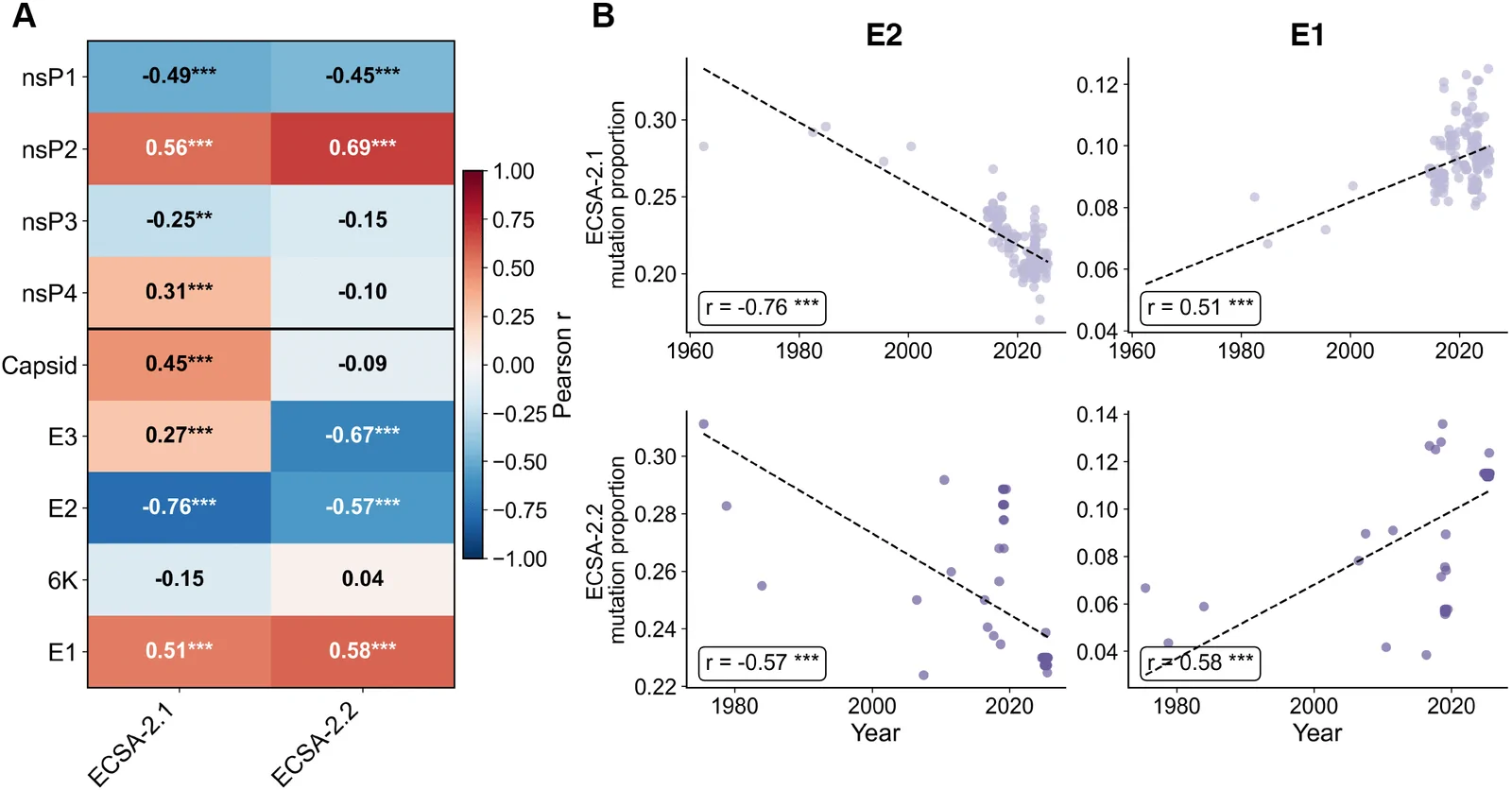
E2–E1 mutation proportion dynamics in emerging lineages. **(A)** Heatmap of protein mutation proportion correlations with molecular clock node time. Red indicates positive correlation; blue indicates negative correlation. Both ECSA-2.1 and ECSA-2.2 showed negative correlations for E2 and positive correlations for E1. Correlations were assessed using Pearson correlation; **P < 0.01, ***P < 0.001. **(B)** Scatter plots of E2 and E1 mutation proportions versus molecular clock time. Top row: ECSA-2.1; bottom row: ECSA-2.2. Dashed lines indicate linear regression fits.

### Distinct vector-associated adaptive strategies accompany regional ECSA dominance

Integrating the preceding analyses of lineage-characteristic substitutions, convergent evolution and selection pressure, we grouped candidate sites by mosquito-adaptive association into *Ae. aegypti*-associated, *Ae. albopictus*-associated and shared ECSA-background classes (S4 Table). In addition to the experimentally validated vector-adaptive substitutions, the table lists new candidates that are supported by genetic evidence but have not been tested experimentally. Within each class, sites were ranked by the number of concordant evidence layers, including convergence, lineage-characteristic status and positive selection. *Ae. aegypti*-associated sites were drawn mainly from ECSA-2.1. Besides the validated substitution E1:K211T, the highest-ranking envelope candidates were E2:A103T, E3:K29R, E1:A305T, E1:A377V and E1:M407L. The strongest nonstructural candidate was nsP3:S330A. *Ae. albopictus*-associated sites were drawn mainly from ECSA-2.2. Besides the validated substitutions E1:A226V and E2:L210Q, additional envelope candidates included E1:T37I, E1:S250P, E1:I317V, E1:K324R and E1:V399I; E2:A246D and E2:Q282K, and 6K:S60N. The highest-ranking nonstructural candidate was nsP3:T337A. Among vector-independent ECSA background sites, the envelope substitutions were E2:A164T and E1:M269V, and the nonstructural substitutions were nsP2:H374Y and nsP3:I376T. These recur across sub-lineages and are more consistent with a shared ECSA molecular background than with adaptation to a single mosquito species. E2:V386A is characteristic of IOL and falls within the high pN/pS window at E2:350–400 in ECSA-2.1. E2:377 and E2:384 in the same interval show only single-method positive-selection signals. These three positions are candidate surveillance markers in the Americas.

A summary of the phylogenetic relationships, vector-associated substitutions and regional transmission patterns shows that differentiated mosquito adaptive strategies underlie the distinct evolutionary trajectories and regional dominance of the three ECSA sub-lineages (Fig 7). ECSA-IOL underwent a temporal shift in vector-associated substitutions. Early IOL carried E1:A226V and E2:I211T and was associated with the *Ae. albopictus*-associated 2005–2006 Indian Ocean epidemic, whereas late IOL carried E2:V264A and E1:K211E and was associated with *Ae. aegypti*-associated transmission in Southeast Asia. In the Americas, ECSA-2.1 carrying E2:V264A and E1:K211T expanded and progressively displaced the Asian genotype, consistent with an *Ae. aegypti*-associated adaptive pattern. ECSA-2.2 independently acquired E1:A226V, E2:L210Q and E2:I211T, showed convergence with early IOL towards *Ae. albopictus*-associated adaptation and was associated with the 2024–2025 multiregional resurgence. Together, these findings suggest that distinct vector-associated adaptive strategies accompanied the regional epidemiological success of the three ECSA sub-lineages.

**Fig 7:**
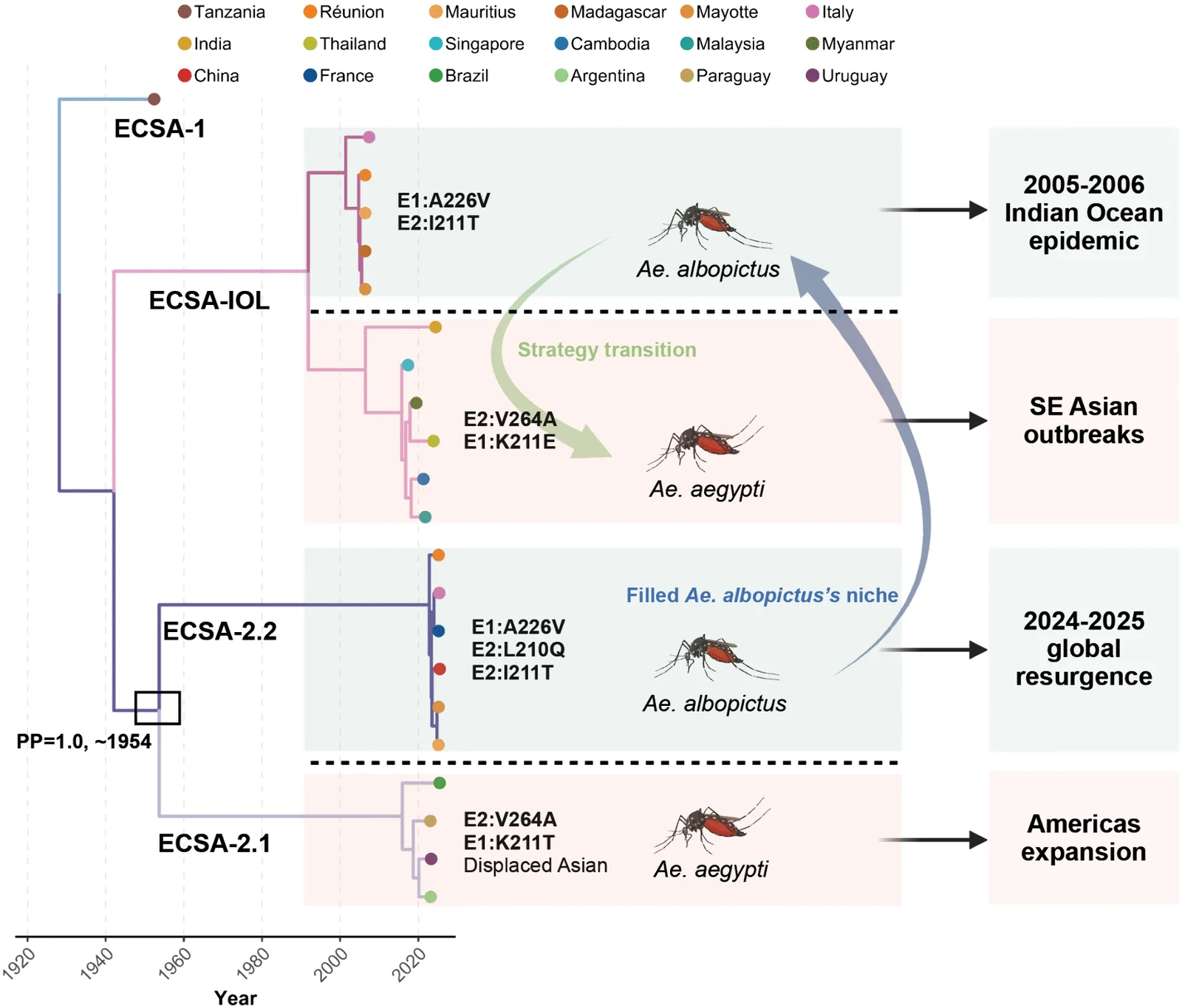
Mosquito adaptive strategies of three ECSA sub-lineages. Time-scaled phylogenetic schematic of ECSA lineages. Tip colors indicate sampling country. Cyan backgrounds mark *Ae. albopictus*–adapted clusters (early IOL and ECSA-2.2). Pink backgrounds mark *Ae. aegypti*–adapted clusters (late IOL and ECSA-2.1). Early IOL carried E1:A226V and E2:I211T during the 2005–2006 Indian Ocean epidemic and later switched to E2:V264A and E1:K211E in Southeast Asian outbreaks. ECSA-2.2 independently acquired E1:A226V, E2:L210Q and E2:I211T, filling the *Ae. albopictus* niche in the 2024–2025 resurgence. ECSA-2.1 carried E2:V264A and E1:K211T during expansion in the Americas and displacement of the Asian genotype. The ECSA-2 split is dated to around 1954 (PP = 1.0).

## Discussion

What determines the epidemiological success of viral lineages? This study found that the fastest-evolving Asian genotype was rapidly replaced in the Americas by slower-evolving ECSA-2.1, while ECSA-IOL’s dominance in the Indian Ocean region gradually yielded to ECSA-2.2. By comparing molecular evolutionary features across three ECSA sub-lineages, we show that the key to lineage success lies in three interrelated factors: accumulation of lineage-characteristic and convergent substitutions, lineage-specific selection pressure redistribution, and differentiated vector adaptation strategies enabling niche partitioning. Together, these findings suggest a molecular evolutionary framework for the global success of ECSA lineages.

### Common molecular basis of ECSA sub-lineage success

The three ECSA sub-lineages (ECSA-IOL, ECSA-2.1, ECSA-2.2) have achieved regional epidemiological success in Southeast Asia, the Americas, and Réunion/China/Italy/France. Whole-genome comparison reveals each sub-lineage accumulated different numbers of lineage-characteristic substitutions. Despite differences in mutation profiles, four core adaptive sites may represent the common molecular basis of ECSA success. The 49 convergent sites indicate common selection pressures faced by ECSA lineages. These sites occurred most frequently in E2 (12) and nsP3 (10), followed by nsP1 (9) and E1 (7), suggesting replication complex assembly and envelope protein function as core adaptive modules. E2’s enrichment aligns with its dual receptor-binding and immune evasion roles [33]. nsP3’s convergent evolution is consistent with its intrinsically disordered region, which maintains function while allowing sequence plasticity [34,35]. Among the three sub-lineages, ECSA-2.2 accumulated the highest number of lineage-characteristic substitutions (49), with enrichment in the E2–E1 receptor-binding region, warranting priority surveillance.

The recurrent emergence of these mutations across independent viral lineages mirrors convergent patterns reported at influenza antigenic sites and in the SARS-CoV-2 spike protein [36,37]. Notably, our convergence analysis recovered all six experimentally validated mosquito-adaptive substitutions, suggesting that recurrent evolution can provide a useful first-pass filter for functionally relevant changes. We next incorporated lineage specificity and positive-selection signals to rank the broader candidate set according to the concordance of multiple evidence layers. Under our proposed model of lineage-specific vector adaptation, mutations characteristic of ECSA-2.1 were classified as putative *Ae. aegypti*-adaptive candidates, whereas those characteristic of ECSA-2.2 were classified as putative *Ae. albopictus*-adaptive candidates. Mutations recurring across sub-lineages were instead assigned to a shared ECSA-background category because their distribution was more consistent with a shared genetic background than with adaptation to a single mosquito species. This framework therefore generates testable hypotheses about lineage-specific vector adaptation, with the highest-ranking unvalidated candidates providing focused targets for reverse-genetics and vector-competence assays. If experimentally validated, these candidates could clarify the molecular basis of differential vector competence and improve assessments of lineage-specific emergence and transmission risk. Additionally, the elevated pN/pS across E2 residues 350–400 in ECSA-2.1 suggests localized adaptive change and identifies this region as a potential focus for genomic surveillance in the Americas.

### Selection pressure redistribution in ECSA-2.1

The fastest-evolving Asian genotype was replaced by slower-evolving ECSA-2.1 in the Americas, which can be partly explained by selection pressure redistribution. Compared to the Asian genotype, which concentrated selection in nonstructural proteins, ECSA-2.1 showed intensified selection in nsP1 and multiple structural proteins (C, E2, 6K, E1). nsP1 is a key component of the alphavirus replication complex responsible for mRNA capping and membrane anchoring [38], with its N-terminal region accumulating multiple sites of significantly enhanced selection, potentially reflecting continuous optimization of replication efficiency within host cells. Among structural proteins, C is responsible for genome packaging, 6K participates in viral budding, E2 mediates receptor binding and cell entry, and E1 mediates membrane fusion and contains key mosquito adaptation sites. Coordinated selection intensification of these proteins suggests a shift in adaptive focus from replication toward virion assembly and transmission.

Further differentiation within ECSA-2.1 suggests direct contributions of mosquito adaptation to lineage spread. Two sub-clusters emerged in the Americas: one confined to Brazil, another spreading to Argentina and Paraguay after fixing the *Ae. aegypti* adaptation site E2:V264A. This pattern suggests E2:V264A acquisition as a key adaptive event for cross-border transmission.

### Mosquito-specific adaptation drives regional lineage success

ECSA-IOL showed a temporal shift in vector-associated substitutions: early IOL, marked by E1:A226V, was associated with *Ae. albopictus* in the Indian Ocean region, whereas late IOL carried the E2:V264A/E1:K211E combination associated with *Ae. aegypti* in Southeast Asia. This switch was accompanied by declining E1:A226V frequency and disappearance of Contrast-FEL-detected selection signals, reflecting ECSA-IOL’s evolutionary trajectory of shifting away from the *Ae. albopictus* niche. This adaptation strategy switch enabled ECSA-IOL to maintain dominance in Southeast Asian regions where *Ae. aegypti* is the primary vector.

ECSA-2.2 independently acquired all three *Ae. albopictus* adaptation sites (E2:L210Q, E2:I211T, E1:A226V) through parallel evolution and caused outbreaks in *Ae. albopictus*-dominant regions including Réunion and Guangdong during 2024–2025. This cross-lineage convergent evolution may indicate that *Ae. albopictus* adaptation represents a strong selective state, with different lineages evolving identical molecular solutions under similar ecological pressures. Notably, ECSA-2.2 also accumulated unique mutations on the E2–E1 complex surface beyond those shared with early IOL, which may participate in receptor binding or immune evasion. Combined with its 49 lineage-characteristic substitutions (highest among the three sub-lineages), ECSA-2.2 may possess stronger adaptive potential and warrants priority surveillance attention. Other co-mutation modules identified in this study may also contribute to this adaptation process.

Two complementary data sources place the molecular evolutionary findings in an ecological context. The 46 mosquito-derived ECSA genomes provide direct vector-source annotations for individual viruses, whereas country-level mosquito-surveillance records document the presence of *Ae. aegypti* and *Ae. albopictus* in regions where the corresponding lineages circulated. Together, the lineage-specific patterns in mosquito-derived genomes and the temporal shift observed within IOL suggest that vector associations are evolutionarily dynamic rather than fixed attributes of individual lineages. Although country-level records do not quantify local vector abundance or transmission contribution, they establish the ecological plausibility of the inferred lineage–vector associations in the relevant geographic settings. Considered alongside the multi-layer candidate ranking, these observations support a testable model in which ECSA-2.1 and ECSA-2.2 may have followed distinct adaptive routes associated with *Ae. aegypti* and *Ae. albopictus*, respectively, potentially contributing to differences in their regional persistence and spread. Temporally and geographically matched mosquito sampling, coupled with reciprocal vector-competence assays, will be required to determine whether the prioritized mutations confer lineage- and vector-dependent fitness effects.

At a broader level, two independent emerging ECSA lineages exhibited consistent E2–E1 mutation dynamics. E2 mutation proportion declined over time while E1 proportion increased. This consistent pattern across independently evolving lineages suggests a temporal shift in adaptive priorities, potentially with receptor binding optimization preceding mosquito vector adaptation, though the underlying mechanism requires further investigation.

Overall, the three sub-lineages formed clear niche differentiation through differentiated mosquito adaptation strategies. ECSA-IOL dominates Southeast Asia, ECSA-2.1 dominates the Americas, and ECSA-2.2 carries *Ae. albopictus* adaptation sites in the Indian Ocean and temperate regions, avoiding direct competition.

### Public health implications

This study identifies several priorities for CHIKV genomic surveillance. ECSA-2.2 merits particular attention because it contains the largest set of lineage-characteristic substitutions (n = 49), including multiple changes in the E2 and E1 envelope proteins, which are involved in viral entry and antibody recognition. Monitoring the geographic spread of ECSA-2.2 will therefore be important, particularly in regions where competent mosquito vectors are established. In the Americas, elevated pN/pS across the E2 C-terminal region of ECSA-2.1 identifies this region as a candidate hotspot of localized adaptive change. The Brazilian sub-cluster should therefore be monitored for additional mutations that could alter vector compatibility or transmission potential. At the site level, the multi-layer ranking provides an evidence-weighted framework for prioritizing surveillance and experimental follow-up. The emergence and frequency trajectories of the highest-ranking putative vector-associated substitutions should be tracked within their lineage backgrounds, while vector-independent ECSA-background substitutions may help define the genetic contexts in which these changes arise. Longitudinal monitoring of these candidate profiles could identify emerging variants that warrant rapid phenotypic assessment. If reproduced in independent datasets, temporal shifts in the relative accumulation of E2 and E1 mutations could provide an additional genomic pattern associated with lineage expansion.

The 2024–2025 Réunion outbreak cluster and closely related viruses detected during the 2025 China outbreak and in travel-associated cases in mainland France and northern Italy illustrate the recent geographic expansion of ECSA-2.2 [5,6,39,40]. In Italy, the 2007 outbreak was caused by an IOL virus carrying E1:A226V, whereas the 2017 outbreak belonged to the late-IOL lineage and lacked this substitution, consistent with previous studies [41]. Our phylogeny also includes a separate ECSA-2.2 genome sampled in Italy in 2017 that lies outside the Réunion outbreak cluster but already carries E1:A226V, E2:I211T and E2:L210Q. This indicates that an ECSA-2.2 genetic background carrying established *Ae. albopictus*-associated substitutions was already present in Europe before the emergence of the recent outbreak cluster. Additional mutations observed within the Réunion cluster may have further influenced viral phenotype. These additional changes, together with other high-priority candidates identified in this study, should be evaluated individually and in combination in the ECSA-2.2 genetic background using reverse-genetics and *Ae. albopictus* vector-competence assays.

Operationally, these findings support linking sequence-based lineage identification to temporally matched vector surveillance. Under our proposed vector-association framework, detection of an expanding ECSA-2.1 lineage in settings where *Ae. aegypti* is locally abundant, or ECSA-2.2 where *Ae. albopictus* is locally abundant, could guide which mosquito populations are prioritized for intensified trapping and infection testing. Local vector abundance, infection prevalence, seasonal activity and epidemiological evidence would then determine the timing and scale of adult and larval control around epidemiologically linked exposure sites. Repeated local transmission in Europe, including hundreds of locally acquired cases in France and Italy in 2025 [7–9], further highlights the need to sequence both imported and autochthonous infections and integrate the resulting lineage information with entomological evidence. Climate change is projected to increase the seasonal suitability for *Ae. albopictus*-borne virus transmission across much of Europe [42], potentially creating greater opportunities for compatible ECSA lineages to establish local transmission.

These findings may also inform the evaluation of vaccine breadth. Two CHIKV vaccines have received regulatory authorization in one or more jurisdictions, although their current availability and recommendations vary: the live-attenuated VLA1553/IXCHIQ, derived from an ECSA-IOL strain, and the virus-like particle vaccine PXVX0317/VIMKUNYA, based on structural proteins from the West African strain 37997 [13,43]. Both vaccines induce cross-neutralizing antibodies against representative strains from the major CHIKV lineages [44,45], supporting broad antigenic conservation. The concentration of lineage-characteristic and convergent substitutions in E2 and E1 identified here nevertheless provides a focused set of contemporary variants for testing this breadth. Neutralization assays using representative ECSA-2.1 and ECSA-2.2 isolates, together with engineered viruses carrying high-priority envelope substitutions, could determine whether vaccine-induced responses remain comparable across the expanding ECSA sub-lineages. The same contemporary virus panel could also support the evaluation of candidate vaccines based on inactivated-virus, viral-vector and mRNA platforms [46]. Together, these experiments would extend genomic surveillance into a practical framework for assessing whether current and future vaccines retain broad coverage as ECSA lineages continue to diversify. This study provides a molecular evolutionary framework for CHIKV lineage success by characterizing three ECSA sub-lineages with distinct regional and vector-associated evolutionary patterns. ECSA-2.1 predominated in the Americas and showed intensified selection on several structural proteins together with *Ae. aegypti*-associated substitutions. ECSA-IOL showed a temporal shift in vector-associated substitutions in Southeast Asia, whereas ECSA-2.2 emerged in the Indian Ocean region and China with convergently acquired *Ae. albopictus*-associated substitutions.

### Limitations

Analyses based on public database sequences may be affected by sampling bias; insufficient sequence coverage in certain regions and time periods may affect the accuracy of mutation-frequency estimates and selection-pressure detection. The site-level mutation analyses focused on amino acid substitutions in protein-coding regions; insertions, deletions, and sequence variation in untranslated regions were not evaluated, and their potential contributions to CHIKV adaptation remain to be investigated. Functional annotation of putative mosquito-associated sites primarily relies on published experimental validation studies; the functional significance of newly identified lineage-characteristic sites and positive selection sites awaits experimental confirmation. Mosquito-derived genomes with explicit vector annotations remain limited and phylogenetically clustered, while country-level vector records indicate species presence rather than local abundance, infection status or contribution to transmission during individual outbreaks. The proposed associations between specific ECSA sub-lineages, candidate mutations and mosquito species should therefore be regarded as testable hypotheses. Additionally, associations between differences in selection pressure and lineage success remain correlational; reverse-genetics and vector-competence experiments in matched lineage backgrounds are needed to validate the adaptive effects of key mutations, both individually and in combination. The proposed E2–E1 temporal pattern requires validation in independent datasets, and the implications of contemporary ECSA substitutions for vaccine breadth should be assessed through neutralization assays.

## Conclusion

This study provides a molecular evolutionary framework for CHIKV lineage success by demonstrating how three ECSA sub-lineages achieve differentiated regional dominance through distinct adaptive strategies. ECSA-2.1 dominates the Americas through intensified structural protein selection and *Ae. aegypti* adaptation. ECSA-IOL maintains success in Southeast Asia via adaptation strategy switching, and ECSA-2.2 emerges in the Indian Ocean and temperate regions through convergent acquisition of *Ae. albopictus*-adaptation sites. Together, these differences support a testable model in which vector-associated ecological conditions contribute to the distinct regional evolutionary trajectories of ECSA sub-lineages. The consistent E2–E1 mutation dynamics observed in emerging lineages may reflect staged adaptation priorities warranting further investigation. These findings provide an evidence-weighted set of candidate mutations for lineage-resolved genomic surveillance and experimental follow-up, and may inform integrated vector surveillance, targeted control planning and the evaluation of vaccine neutralization breadth.

## Materials and methods

### Data collection and sequence processing

CHIKV whole-genome sequences were downloaded from the GISAID EpiArbo database (accessed October 13, 2025) [47]. Multiple sequence alignment was performed using MAFFT v7.490 [48] --addfragments mode, with EPI_ISL_17462250 (1952 Tanzania isolate) as the reference sequence, using the --keeplength parameter to maintain reference sequence length. Screening criteria were complete or near-complete sequences with genome length >10,000 nt and alignment gap ratio ≤1%. After quality control, 4,120 CHIKV whole-genome sequences were retained for downstream analysis (sequence accession numbers and genotype assignments are provided in S1 Data). Epidemiological data on CHIKV transmission history were sourced from WHO surveillance reports and published literature (S2 Data). Genotype and ECSA sub-lineage assignment are described below.

Public sequence databases are subject to geographic and temporal sampling heterogeneity, with potential overrepresentation of intensively surveilled outbreaks and underrepresentation of regions such as sub-Saharan Africa. To mitigate these biases, several strategies were employed across downstream analyses, including clustering of highly similar sequences to reduce redundancy, hierarchical down-sampling by genotype, country, and year, and removal of identical sequences prior to selection pressure detection, thereby improving the balance of sequence representativeness and reducing the interference of redundant sampling on statistical inference.

### Maximum likelihood phylogenetic analysis

Maximum likelihood (ML) phylogenetic trees were constructed using IQ-TREE v2.2.0 [49] with ModelFinder automatic model selection. GTR + F + R10 was selected as the best substitution model based on Bayesian Information Criterion (BIC). The O’nyong-nyong virus sequence (NC_075006.1) was used as outgroup to construct the rooted tree, with 1,000 ultrafast bootstrap replicates to assess branch support. Phylogenetic trees were visualized and annotated using iTOL v6 [50].

### Evolutionary rate estimation

Evolutionary rate estimation for each major genotype (West African, Asian, ECSA-IOL, ECSA-2) was performed using TreeTime v0.11.3 [51]. Except for the West African genotype, sequences from the other three lineages were clustered using CD-HIT v4.8.1 [52] (sequence-identity threshold, 0.999) before analysis. TreeTime used GTR substitution model and joint likelihood optimization for root-to-tip regression analysis; R² values for the four lineages were 0.90 (West African), 0.99 (Asian), 0.76 (ECSA-IOL), and 0.87 (ECSA-2), indicating good molecular clock signal. ECSA-1 was omitted from this analysis. Only 22 genomes were available for this lineage, of which seven lacked collection dates, and the root-to-tip temporal signal was too weak for reliable rate estimation.

### Bayesian molecular clock analysis

Bayesian phylogenetic analysis was performed using BEAST v1.10.5 [53] to infer divergence times. Hierarchical downsampling selected 726 representative sequences: first ensuring at least one sequence coverage for all sampling years and countries of each genotype, then randomly supplementing each genotype-country-year stratum to a maximum of three sequences (10 for ECSA-2.1 and ECSA-2.2 to retain more genetic diversity). The analysis used the HKY + G4 substitution model, uncorrelated relaxed molecular clock (lognormal distribution), and Constant Size Coalescent tree prior, with MCMC chain length of 2 × 10^8^ generations (sampling every 10,000 generations, first 10% as burn-in), most recent sampling date was 2025.6025. Convergence was assessed using Tracer v1.7.2 [54], with trace plots confirming stationarity after burn-in. TreeAnnotator was used to summarize the maximum clade credibility tree with median node heights; node ages were expressed as 95% HPD intervals. Time trees were visualized using R packages treeio and ggtree [55].

### Mosquito adaptation site tracking

Six literature-reported vector-associated amino acid substitutions were tracked on the phylogenetic tree, including substitutions associated with *Ae. albopictus* (E2:L210Q, E2:I211T, and E1:A226V) and *Ae. aegypti* (E2:V264A, E1:K211E, and E1:K211T). Amino acid states at these sites were extracted from all 726 sequences in the BEAST time tree and visualized as heatmaps alongside the time-calibrated phylogenetic tree.

### ECSA sub-lineage assignment

ECSA genomes were first placed on the global maximum-likelihood tree and assigned to ECSA-1, ECSA-IOL or ECSA-2, following the ECSA nomenclature used in genomic studies of the 2024–2025 Réunion re-emergence [5]. ECSA-2 was then divided on the subsampled Bayesian time tree (n = 726) into two sister clades, ECSA-2.1 and ECSA-2.2. The split received maximum posterior support (PP = 1.0) and was dated to July 1954 (95% HPD, August 1948 to February 1958). Genomes were assigned to either clade only when they fell in the corresponding monophyletic group with this support. The division matched geography. ECSA-2.1 was almost restricted to the Americas. ECSA-2.2 occupied Africa earlier and later accounted for outbreaks in Réunion, China and parts of Europe. Early and late IOL were likewise defined from the time tree within ECSA-IOL. Late IOL is a monophyletic clade nested inside ECSA-IOL and consists mainly of sequences from Southeast Asian outbreaks after 2010. The remaining IOL genomes form a paraphyletic group and derive mainly from the 2005–2006 Indian Ocean epidemic. Subsequent marker, selection and vector analyses used this tree-based partition.

### Lineage-characteristic substitution identification

Lineage-characteristic substitutions were identified by comparing all sequences from each ECSA sub-lineage (ECSA-IOL, ECSA-2.1, ECSA-2.2) with the ancestral ECSA sequence (EPI_ISL_17462250, 1952 Tanzania isolate). Amino acid sites with frequency >75% within a sub-lineage and different from ancestral state were defined as lineage-characteristic substitutions, excluding sites conserved in Asian and West African genotypes to focus on ECSA-specific adaptations.

### Convergent evolution

Convergent amino acid substitution sites among ECSA sub-lineages were identified using sitePath v1.14.0 [56]. Based on ECSA branches of the ML phylogenetic tree and corresponding amino acid multiple sequence alignments, parallel mutations independently occurring on at least two lineage paths within ECSA were detected by defining lineage paths. Screening criteria included: sites must have mutations in at least two of three ECSA sub-lineages, with at least 5 sequences carrying the mutation in each sub-lineage; inherited mutations with overall mutation frequency exceeding 90% across all sequences were excluded. After screening, 49 convergent sites were retained. To visualize mutation distribution, sequences from each sub-lineage were clustered for redundancy removal using CD-HIT v4.8.1 at 99.9% identity, then representative sequences were selected using a greedy algorithm, prioritizing sequences covering the most convergent sites (ECSA-IOL: 40, ECSA-2.1: 40, ECSA-2.2: 20). The convergent mutation matrix was finally visualized as a heatmap, with rows representing convergent sites organized by protein domain, columns representing sequences grouped by sub-lineage with accompanying phylogenetic tree, and mutant amino acids colored by physicochemical properties.

### Ancestral sequence reconstruction

Ancestral sequence reconstruction using the method built into Augur v24.3.0 [57] inferred ancestral sequence states at each internal node based on the ML phylogenetic tree of all 4,120 sequences, for subsequent Asian-to-ECSA-2.1 branch mutation analysis.

### Selection pressure

Selection pressure analyses were based on the global ML tree of 4,120 sequences. Each protein-coding region was extracted separately and identical sequences removed before analysis using HyPhy v2.5 [58] command-line tools. RELAX [59] was applied to compare selection intensity differences between lineages, with selection intensity parameter *K* > 1 indicating intensification and *K* < 1 indicating relaxation, using ECSA-2.1 as test group and Asian as reference group (LRT, *P* < 0.05). BUSTED [60] was used to detect gene-level positive selection evidence, with separate analyses for Asian and ECSA-2.1 lineages. Site-level selection used three complementary methods: FEL, MEME, and SLAC [61,62]; sites detected by ≥2 methods with *P* < 0.05 were defined as positive selection consensus sites. Contrast-FEL [63] was applied to identify sites with differential selection pressure between lineage pairs, including ECSA-2.1 vs. Asian and early IOL vs. late IOL; significance was assessed through built-in Benjamini-Hochberg FDR correction (*Q* < 0.05).

### Sliding window pN/pS analysis

To visualize genome-wide selection pressure distribution, sliding window pN/pS ratios were calculated using the Nei-Gojobori method [64]. Analysis windows were 15 codons with step size of 1 codon; Pairwise pN and pS values were calculated for each window, and their ratio was reported as pN/pS. Results were smoothed using a three-window rolling average before visualization.

### Co-mutation module analysis

Mutation frequency dynamics were tracked through sliding window analysis of all ECSA-IOL sequences (n = 758) along evolutionary distance to characterize temporal dynamics of mosquito adaptation strategy switching. First, dynamic sites with mutation frequency change >75% along evolutionary distance were screened; then pairwise Pearson correlation coefficients of mutation states (0/1) were calculated for each site; site pairs with |r| > 0.95 were identified as co-mutation modules, with the correlation matrix visualized as a hierarchically clustered heatmap.

### Structural mapping

Envelope protein mutations were mapped to the CHIKV E2–E1/MXRA8 complex structure (PDB ID: 6JO8) [65] using PyMOL v2.5, with mutations color-coded by lineage origin and functional category.

### Lineage–vector association

Mosquito-derived genomes were identified from sequence annotations as *Ae. aegypti* or *Ae. albopictus* and assigned to early IOL, late IOL, ECSA-2.1 or ECSA-2.2 on the basis of the tree-based partition. Fifty-three CHIKV genomes with explicit mosquito-source annotations were identified, of which 46 ECSA genomes were assigned to early IOL, late IOL, ECSA-2.1, or ECSA-2.2 and retained for the two lineage-pair comparisons. Country-level occurrence records of *Ae. aegypti* and *Ae. albopictus* were obtained from a published global occurrence database [28]. A country was classified as *Ae. aegypti* only, *Ae. albopictus* only, or both, according to whether records of each species were present. Genome sequences were mapped to these classes by sampling country. Countries with records of both species were excluded from the association analysis to limit sympatric confounding.

### Protein mutation proportion analysis

Based on the BEAST time tree of 726 sequences, mutation proportion for each protein was calculated for each sequence (number of amino acid differences in a given protein / total amino acid difference across all proteins in that sequence); this metric reflects each protein’s relative contribution to total mutation load. Pearson correlation coefficients between all protein mutation proportions and node time were calculated separately for each lineage (ECSA-2.1, ECSA-2.2, ECSA-IOL, Asian).

### Statistical analysis and visualization

Pairwise differences in TreeTime evolutionary rates among the West African and Asian genotypes and the ECSA-IOL and ECSA-2 lineages were assessed using a two-sided *z*-test based on the rate estimates and their one-standard-deviation uncertainties reported by TreeTime. Six pairwise comparisons were performed, and the resulting *P* values were adjusted using the Bonferroni method. Among mosquito-derived genomes, associations between mosquito species and either ECSA-IOL period or ECSA-2 sub-lineage were assessed using two 2 × 2 Fisher’s exact tests (early versus late IOL and ECSA-2.1 versus ECSA-2.2), with Holm correction. After excluding genomes sampled in countries with records of both *Ae. aegypti* and *Ae. albopictus*, the association between sampling-country vector-occurrence class and lineage among genomes was assessed using a chi-square test on a 2 × 5 contingency table. Pearson correlation analysis and likelihood-ratio tests implemented in RELAX and BUSTED were performed as described in the corresponding sections. The Benjamini–Hochberg procedure was used to control the false discovery rate in the Contrast-FEL analyses. Analyses were performed using Python v3.10 and R v4.3.0. Statistical significance was defined as *P* < 0.05; for analyses involving multiple-testing correction, adjusted *P* or *Q* values < 0.05 were considered significant unless otherwise stated. Visualizations were generated using ggplot2, ggtree, matplotlib, and seaborn.

### Data and code availability

All CHIKV genome sequences analyzed in this study were obtained from GISAID EpiArbo. Accession numbers and genotype assignments for all 4,120 sequences are provided in S1 Data. The first-outbreak dates and epidemic phases across 119 countries and territories are provided in S2 Data. Analysis pipelines, custom scripts, and visualization code are available at https://github.com/wuaipinglab/chikv-phylogenetics.

## Supporting information

S1 Fig Complete evolutionary node divergence times of ECSA lineage. Bayesian molecular clock timetree with complete node annotations based on 726 sequences. Branch colors: West African (cyan), Asian (yellow), ECSA-1 (blue), ECSA-IOL (pink), ECSA-2.1 (light purple), ECSA-2.2 (dark purple). Blue translucent bars indicate 95% HPD intervals for all well-supported internal nodes. Red dots mark major clade nodes (≥100 descendant tips) with median divergence times and 95% HPD intervals labeled.
(TIF)

S2 Fig Genotype, geographic distribution, and mosquito adaptation mutations on complete phylogenetic tree. (A) Maximum likelihood tree based on 4,120 sequences, colored by genotype: West African (cyan), Asian (yellow), ECSA-1 (blue), ECSA-IOL (pink), ECSA-2.2 (dark purple), ECSA-2.1 (light purple). (B) Same tree colored by country: Réunion (green), China (pink), Brazil (light green), Thailand (purple), India (cyan), USA (blue), Argentina (brown). (C) Distribution of six key mosquito-adaptation sites, shown in five subplots. E1:A226V emerged independently in early IOL and ECSA-2.2; E2:V264A mainly appeared in late IOL and some ECSA-2.1, representing multiple convergent evolution events.
(TIF)

S3 Fig Phylogenetic distribution of four ECSA shared core adaptive sites. Distribution of nsP2:H374Y, nsP3:I376T, nsP4:Q500L, and E2:A164T across the ECSA clade (visualized using the single plot function in SitePath package). All four sites show >90% fixation frequency in three sub-lineages, representing common molecular basis of ECSA success.
(TIF)

S4 Fig Detailed analysis of ECSA-2.1 replacement of Asian in South America. (A) Molecular clock time tree of South American sequences. Branch colors: Asian (yellow), ECSA-2.1 (purple), showing clear temporal genotype replacement pattern. (B) Genotype replacement dynamics. Stacked area plot showing Asian and ECSA-2.1 frequency changes; ECSA-2.1 rapidly dominated after ~2016. (C) Contrast-FEL genome-wide distribution. Δβ (ECSA-2.1 − Asian) indicates selection pressure differences. Yellow: stronger Asian selection; purple: stronger ECSA-2.1 selection. This panel shows all significant sites (P < 0.05); the 16 sites shown in main Fig. 4d are those significant after FDR correction. (D) ECSA-2.1 internal differentiation. Green shading marks sub-cluster with fixed E2:V264A, which spread to multiple South American countries.
(TIF)

S5 Fig Dynamic sites and co-mutation modules in ECSA-IOL adaptation strategy switching. (A) Frequency changes of 17 dynamic sites along evolutionary distance. X-axis: evolutionary distance from IOL MRCA; Y-axis: mutation frequency. Most sites rise from 0% to >80%; E1:A226V shows rise-then-decline reversion trajectory. (B) Pearson correlation heatmap of 17 dynamic sites. Hierarchical clustering reveals multiple co-mutation modules. E1:226 and E2:264/E1:211 locate in different modules, showing mutual exclusivity of Ae. albopictus and Ae. aegypti adaptation markers. (C) Co-mutation module legend showing composition of five co-mutation modules and independent sites.
(TIF)

S6 Fig Association between ECSA sub-lineages and mosquito vectors. (A) Vector composition of 53 CHIKV genomes with explicit mosquito-source information. Stacked bars show the proportions of genomes derived from Aedes albopictus or Aedes aegypti, with numbers indicating genome counts. (B) Of these genomes, 46 belonging to early IOL, late IOL, ECSA-2.1, or ECSA-2.2 were included in the lineage-level analysis. The heatmap shows log_2_(O/E), where O and E denote the observed and expected counts, respectively. Associations between mosquito species and lineage group were assessed using two prespecified two-sided Fisher’s exact tests (early versus late IOL and ECSA-2.1 versus ECSA-2.2), with Holm correction. (C) Amino acid states at 18 literature-reported vector-associated sites among these 46 mosquito-derived genomes. Based on experimental evidence from previous studies, the sites were classified as substitutions enhancing adaptation to Ae. albopictus, Ae. aegypti, or both vector species. The supporting literature and experimental evidence are provided in S3 Data. Sequences are grouped by lineage and mosquito source, with sampling years indicated. E2:L210Q was not detected in these sequences. (D) Countries classified according to global mosquito occurrence data as having records of Ae. aegypti only, Ae. albopictus only, or both species. Numbers above the bars indicate the number of countries in each category. (E) Numbers of Ae. aegypti and Ae. albopictus occurrence records in key countries. Bar colors represent log_10_(n + 1)-transformed occurrence counts, where n is the number of records. Numbers above the bars indicate the corresponding untransformed counts. (F) Lineage distributions of 442 CHIKV genomes with sampling-country information after excluding countries with records of both mosquito species. The heatmap shows relative enrichment or depletion as log_2_(O/E). The overall association between lineage composition and country-level vector category was assessed using a χ² test (χ² = 162.71, df = 4, P = 3.83 × 10^-34^).
(TIF)

S7 Fig Protein mutation proportion temporal dynamics in ECSA-IOL and Asian. (A) Heatmap of protein mutation proportion correlations with molecular clock time. Red indicates positive correlation; blue indicates negative correlation. ECSA-IOL E1 shows positive correlation with increasing trend, while Asian E1 shows negative correlation with decreasing trend. **P < 0.01, ***P < 0.001. (B) Scatter plots of E2 and E1 mutation proportions versus molecular clock time. Top row: ECSA-IOL; bottom row: Asian. Dashed lines indicate linear regression fits.
(TIF)

S1 Table Abbreviations used in this study. (XLSX)

S2 Table Lineage-characteristic substitutions in three ECSA sub-lineages. (XLSX)

S3 Table Convergent and lineage-characteristic substitutions across ECSA sub-lineages. (XLSX)

S4 Table Evidence-ranked candidate substitutions grouped by mosquito-adaptive class. (XLSX)

S1 Data CHIKV genome sequence accession numbers and genotype assignments (4,120 sequences). (XLSX)

S2 Data CHIKV first-outbreak dates and epidemic phases across 119 countries and territories. (XLSX)

S3 Data Experimentally validated mosquito-adaptive mutations reported in the literature. (XLSX)
